# Optimizing the Coagulation Potential of Pineapple (*Ananas comosus*) Extract for Chickpea Cheese Production

**DOI:** 10.1002/fsn3.71381

**Published:** 2025-12-26

**Authors:** Lamrot Damene Alemayehu, Eskindir Getachew Fentie, Henock Woldemichael Woldemariam

**Affiliations:** ^1^ Department of Chemical Engineering, College of Engineering Addis Ababa Science and Technology University Addis Ababa Ethiopia; ^2^ NGS Center Kyungpook National University Daegu Korea; ^3^ Center of Excellence for Biotechnology and Bioprocess Addis Ababa Science and Technology University Addis Ababa Ethiopia; ^4^ Center for Oceanography, Costal Engineering and Neritic Sciences, College of Science, Engineering and Technology University of South Africa Johannesburg South Africa

**Keywords:** chickpeas, coagulant, pineapple extract, plant‐based cheese

## Abstract

In recent years, the market demand for plant‐based cheese analogue is growing. This study aimed on the development and characterization of chickpea cheese coagulated with pineapple ( *Ananas comosus* ) extract. Box–Behnken design was employed to optimize the experimental conditions. According to the experimental design, the optimum point was obtained at 0.24 milk to extract ratio, 40°C coagulation temperature and 58.72 min coagulation time. The yield, hardness, cohesiveness, and adhesiveness, L*, a* and b*, and pH of chickpea cheese using the optimized formula were estimated to be 27.96%, 35.25 N, 0.59 N, 2.04 N, 81.83, 1.91, 30 and 5.74 respectively. Pineapple ( *Ananas comosus* ) extract showed high milk clotting activity U/mL (50.18 ± 0.3 U/mL), inhibition zone against *Staphylococcus aureus* (13.99 ± 0.02) and *Escherichia coli* (6.45 ± 0.05). As the DPPH and ABTS assays pineapple extract exhibited notable antioxidant activity with IC50 values of 233.021 and 227.33 μg/mL, respectively. The yield, hardness, cohesiveness, and adhesiveness, for optimized cheese (27.54% ± 0.1%, 35.04 ± 0.01 N, 0.57 ± 0.01 N, 2.14 ± 0.01 N) and (18.89% ± 0.13%, 24.22 ± 0.01 N, 0.29 ± 0.01 N and 1.68 ± 0.01 N) for control cheese (chickpea cheese without pineapple ( *Ananas comosus* ) extract) was observed, respectively. The protein content of optimized cheese was 9.77 ± 0.05 whereas the control cheese was 6.2 ± 0.03. Addition of pineapple extract significantly increased (*p* > 0.05) the nutritional composition (Potassium, Magnesium, and Calcium) of optimized cheese. The sensory properties for optimized cheese were 4.5 for appearance and aroma; 4.4 for texture, taste and overall acceptability while the control cheese obtained 3.2 for appearance, 3.1 for aroma, 2.8 for texture, 3.9 for taste and 2.7 for overall acceptability. Generally, the study offers innovative utilization of pineapple ( *A. comosus* ) extract as a natural coagulant to develop chickpea cheese offering a nutritious and sustainable option that aligns growing demand for plant‐based dairy products.

## Graphical abstract

This study developed and optimized chickpea‐based cheese using pineapple ( *Ananas comosus* ) extract as a natural coagulant. The optimized formulation improved yield, texture, protein content, mineral composition, and sensory acceptability compared to control cheese. Findings highlight pineapple extract's clotting, antimicrobial, and antioxidant properties, offering a nutritious and sustainable plant‐based cheese analogue that meets growing consumer demand.

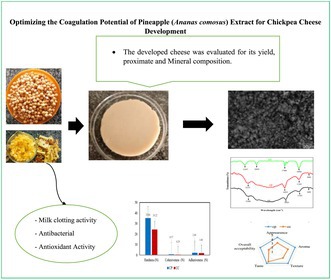

## 1 Introduction

The growing awareness about nutrition and health, humane production of food, and sustainability are conductive factors for the popularity of plant‐based foods (Udayarajan et al. 2022). In addition, moving toward eating plant‐based foods contributes toward reducing greenhouse gas emissions (Grasso et al. 2021). Despite their textural and lower nutrient bioavailability than dairy cheese, the global plant‐based food market is projected to grow $37.45 billion by 2030 at CARG of 10.65%, and the vegan cheese market is projected to reach $7.10 billion at a CAGR of 12.6% (Grand View Research 2022).

Within the plant‐based food market, plant alternatives to cheese are expected to grow a value of nearly $4 billion by 2024 as cheese is one of the most cherished foods in many parts of the world (Grasso et al. 2021). To meet the cheese demand, plant‐based products that are based on grains, seeds, nuts, fruits, and other plant products have appeared in the mainstream market (Craig et al. 2022). According to Clegg et al. (2021) the growing acceptance and adoption of plant‐based diary alternatives (PBDAs) has grown exponentially in recent years.

In recent years, plant‐based dairy alternatives, including cheese, have entered the food market as alternative protein options for ordinary consumers, vegetarians, and consumers with lactose intolerance (McClements et al. 2019). According to Reyes‐Jurado et al. (2023), cheese is a solid product which is obtained by curdling milk with rennet.

Pulses can offer opportunities for novel food product development and contribute to achieving recommended daily protein requirements (Roy et al. 2010). In particular, chickpeas are considered very nutrient‐dense, containing 20% protein (Hall et al. 2017). As they are nutritious (rich in protein, minerals, cholesterol‐free) and have a less beany taste in flavor profile, chickpeas show great potential in the development of new and reformulated food products (Withana‐Gamage et al. 2011). In light of the economic viability of chickpea cultivation and its substantial resources, its inclusion in the production cycle as a valuable source of vegetable protein is significant (Pozhitkova 2020).

The coagulation of chickpea milk is done by coagulating the soluble protein in the milk with the aid of various coagulants. Specifically, plant‐derived coagulants have become a subject of growing interest in the cheese industry due to their availability and easy purification process (Fguiri et al. 2021). Pineapple ( *Ananas comosus* ), containing bromelain enzyme which hydrolyzes protein, promotes curd formation and potential to offer antioxidant properties and improved cheese texture (Mäkinen et al. 2016).

The aim of this study was the development and characterization of chickpea cheese by coagulating the milk with pineapple ( *A. comosus* ) extract. Prior to developing the cheese, through ultrasonic‐assisted extraction (UAE) pineapple ( *A. comosus* ) extract was prepared and chickpea milk was extracted. The developed cheese was evaluated for its yield, proximate composition, physicochemical, structural, microbiological, morphological, nutritional, and sensory properties of the developed chickpea cheese.

## 2 Materials and Methods

### 2.1 Sample Collection and Preparation

The chickpea (*Kabuli* variety) utilized in this study was obtained from the Awash Melkasa Agricultural Research Center (AMARC), Ethiopia. The fresh mature golden yellow skin pineapple fruit was obtained from the local market in Addis Ababa, Ethiopia, whereas nettle and pawpaw leaf were generously collected by nearby farmers (around Koye Feche, Addis Ababa). The collected samples were packed in a polyethylene plastic bag and transported to the Addis Ababa Science and Technology University laboratory. Then the collected samples were stored at refrigerated conditions (4°C) till further analysis.

### 2.2 Processing Methods

#### 2.2.1 UAE Assisted Coagulant Extraction Process

**FIGURE 1:**
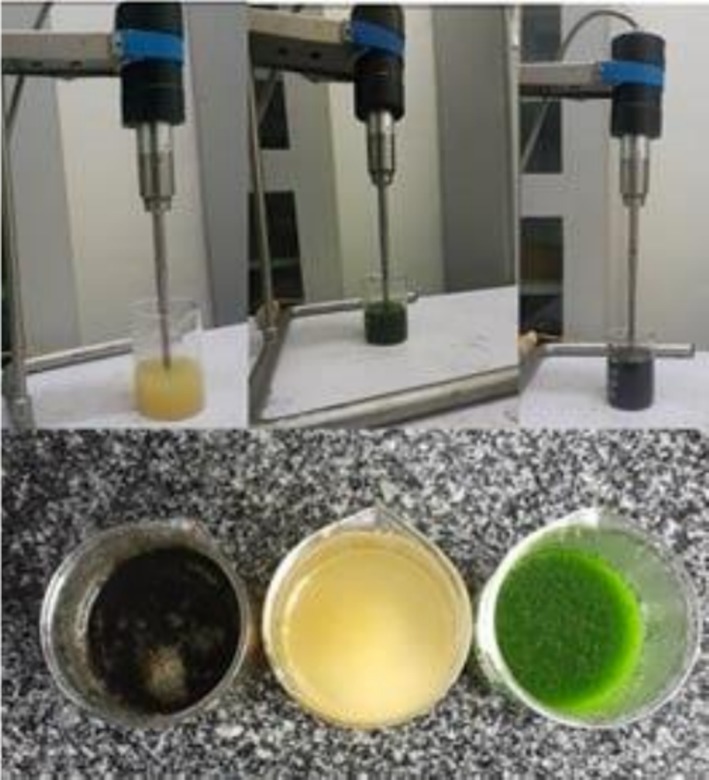
Ultrasonic extraction of the selected plants.

The extraction process was carried out following the method described by Luchian et al. (2023). Ultrasound‐assisted extraction (SJIA‐950 W, Ningbo, Sjila Lab Equipment Co. Ltd., China) was employed to extract the liquid extract obtained from pineapple ( *Ananas comosus* ), and the other two plant sources, including nettle and papaw leaves as shown in Figure 1. Distilled water was used as a solvent (20 mL/2 g ratio). A probe with a diameter of 6 mm, power of 75 W, and frequency of 20 kHz was utilized for the UAE process, which lasted for 30 min. The ultra‐sonication was performed at room temperature (25°C) without active temperature control using a water bath or other external controlling system.

After the extraction process was completed, the sample was immediately cooled to room temperature (20°C) and then subjected to centrifugation (FUNKE GERBER 12106 Berlin, Germany) at a speed of 1200 rpm for 15 min. The supernatant was filtered using filter paper to separate the liquid extract from the solid plant material. The solid plant material was discarded and the filtrate was stored at −20°C until further use.

#### 2.2.2 Chickpea Milk Processing

**FIGURE 2:**
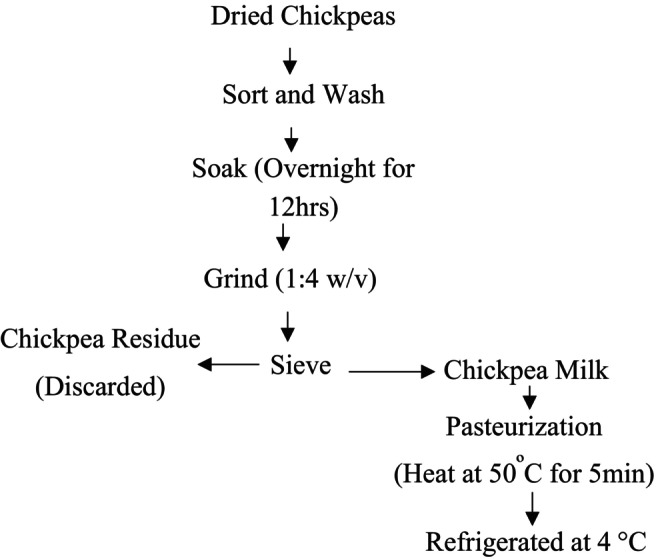
Procedure for Chickpea Milk Preparation (*Source:* Omueti and Jaiyeola (2006)).

Chickpeas were washed and soaked in distilled water overnight to assure proper hydration of the chickpeas, which leads to ease of transformation to a smooth paste upon grinding. The soaked chickpeas were blended by stopping the blender periodically to push down the sides so that no unblended parts remained and brought the pest in to a beaker. The chickpea paste was diluted with distilled water (4:1), and milk was filtered using cheese cloth and strained into a container. Chickpea milk was pasteurized by heating at 50°C for 5 min on a plate. During heating, the temperature was monitored with a thermometer, and the milk was stirred continuously to prevent scorching and probably over boiling. The heated milk was removed from the hot plate and placed in a water bath to cool, then transferred to a clean container and stored in the refrigerator until analysis. The procedure and steps of chickpea milk preparation are summarized in Figure 2 below.

#### 2.2.3 Procedure for Making Chickpea Cheese

**FIGURE 3:**
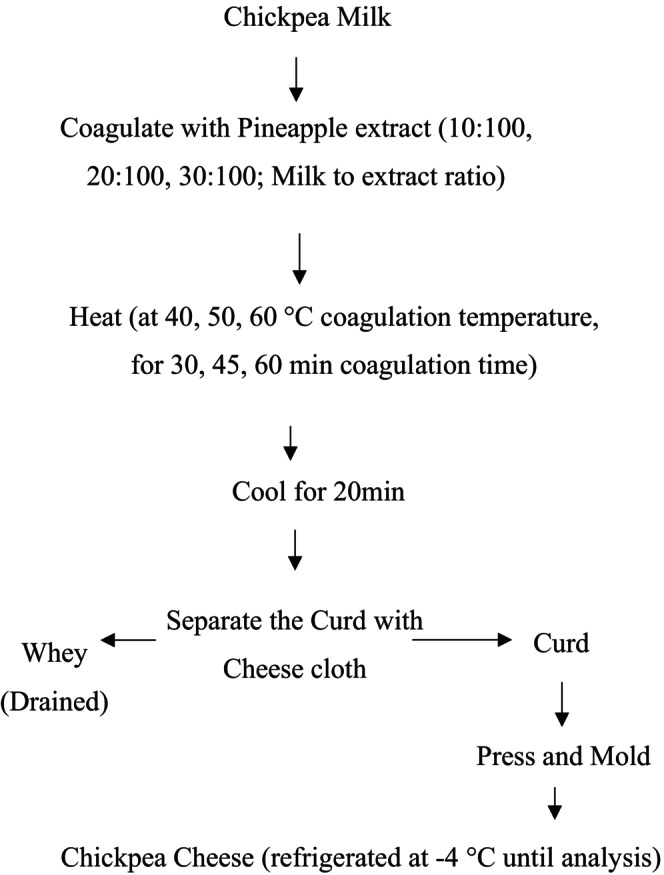
Procedure of Chickpea Cheese Preparation (*Source:* Omueti and Jaiyeola (2006)).

For each experiment, chickpea milk and the extract were mixed in (10:100, 20:100, 30:100; Milk to extract ratio) and incubated at 40°C, 50°C, 60°C coagulation temperature for 30, 45, 60 min coagulation time. Then each of the samples was filtered using cheese cloth to separate the curd from the whey (non‐coagulated part of the mixture). The cheese samples were then pressed for 30 min so that uniformity of the product is assured. The control cheese was produced with no extract with the aid of temperature following the same procedure (the protein and starch in chickpeas can exhibit thickening behavior with the aid of heating). The preparation procedures of chickpea cheese are summarized in Figure 3 below.

### 2.3 Experimental Design

The individual and interaction effect of the three independent variables of A (Milk‐Extract ratio), B (Coagulation temperature) and C (Coagulation time) on yield, texture (hardness, cohesiveness, and adhesiveness), color (L*, a*, b*) and pH was investigated using Response Surface Methodology (Box–Behnken design) with 17 runs randomized order. The aim of optimizing formulation of chickpea cheese was to maximize yield, hardness, cohesiveness, adhesiveness, L*, and b*, and to minimize, a*, and pH.

The processing variables (Table 1) of this study were chosen since it has been reported by many studies that the coagulation parameters strongly affect the important parameters of cheese (Ghamgui et al. 2021).

**TABLE 1:** Levels of factors in Box–Behnken design.

Factors	Name	Unit	Levels		
			−1	0	1
A	Milk‐extract ratio	(mL/mL)	10:100	20:100	30:100
B	Coagulation temperature	(°C)	40	50	60
C	Coagulation time	(min)	30	45	60

*Note:* The parameter rationale was chosen to achieve desirable curd texture and yield as observed in preliminary sensory evaluation.

### 2.4 Methods of Experiment

#### 2.4.1 Yield Determination

##### 2.4.1.1 Yield of Chickpea Milk and Pineapple Extract

Percentage yield of chickpea milk and pineapple extract was calculated using Equations (1a and 1b), respectively. Beakers were pre‐washed with tap water and oven‐dried for 10 min. For the chickpea milk, the yield was determined according to (Onuorah et al. 2007) using Equation (1a).

(1a) $$\%\text{Yield}=\frac{\text{weight of chickpea milk}}{\text{weight of slurry}}\times 100$$

The percentage yield of pineapple extract was determined by measuring the weight of chopped pineapple pieces (*W*_p_) and the liquid extract obtained after UAE (*W*_e_).

(1b) $$\%\text{Yield}=\frac{W_{e}}{W_{p}}\times 100$$

where *W*_e_ = weight of extract, *W*_p_ = weight of pineapple pieces.

#### 2.4.2 Physicochemical and Functional Properties of Pineapple Extract

##### 2.4.2.1 pH

The pH of extract was measured with a calibrated digital pH meter (HI98194). After adjusting the milk to room temperature in a beaker, the electrode was immersed, and the reading was recorded once stabilized.

##### 2.4.2.2 Total Soluble Solids

TSS in chickpea extract was measured using a refractometer following AOAC (2016). The device was calibrated with distilled water, then a drop of milk was placed on the prism, and the Brix percentage reading was recorded.

##### 2.4.2.3 Milk Clotting Activity

Preliminary test was performed to compare Milk Clotting Activity (MCA), a key property of coagulants for pineapple extract, with pawpaw and nettle leaf extracts using the diary federation formula (Equation 2). 10 mL of each extract was added to 100 mL of chickpea milk pre‐incubated at 50°C for 30 min. After stirring for even distribution, coagulation time (the time interval until first curd flakes appeared) was recorded (Table 2).

(2) $$\mathrm{MCA}\;\left(U/\mathrm{mL}\right)=\frac{2400\times V_{m}}{T\times V_{e}}$$

where MCA = milk clotting activity in units/mL of extract; 2400 is a constant (1 unit is defined as the amount of coagulant that clots 2 mL of milk in 2400 s); *V*_m_ = volume of the milk sample (mL); *V*_e_ = volume of coagulant (mL); *T* = coagulation time (seconds).

**TABLE 2:** Formulation of milk to plant extracts for MCA test.

Ingredients	Plant samples		
	PA	NL	PL
Coagulants (mL)	10	10	10
Chickpea milk (mL)	100	100	100

Abbreviations: NL, nettle leaf extract; PA, pineapple extract; PL, pawpaw leaf extract.

##### 2.4.2.4 Antimicrobial Activity

Antimicrobial assays were carried out according to (Ogunmefun et al. 2018). Agar well diffusion method on Mueller‐Hinton agar was used to test susceptibility of *Escherichia coli* and *Staphylococcus aureus* to pineapple extract. The media was sterilized at 121°C for 15 min, poured into plates, solidified, and inoculated by evenly spreading bacterial cultures. The pineapple extract was sterilized through a 0.22 μm filter before application. Plates were incubated at 37°C for 24 h, after which inhibition zones were measured and recorded.

##### 2.4.2.5 Antioxidant Activity

The pineapple extract was assessed for its antioxidant activity involving two distinct methods: DPPH and ABTS assay. For both assays, Ascorbic acid (C6H8O6, 99.9%) was taken as a positive control.

##### 2.4.2.6 DPPH Radical Scavenging Activity

Antioxidant activity of Pineapple extract was assessed for its Scavenging ability on 2, 2‐diphenyl‐1‐picrylhydrazyl (DPPH) radicals following the method of (Yuris and Siow 2014). The DPPH radical scavenging ability of pineapple extract was assessed by preparing a 100 μg/mL stock solution, serially diluted to 20–100 μg/mL with ethanoic DPPH. Solutions were incubated in the dark at room temperature for 30 min, then absorbance was measured at 517 nm using a UV–Vis spectrophotometer (JASCO V‐770, Japan) against an ethanol blank. Radical scavenging activity (RSA) was calculated using Equation (3) based on absorbance values. According to Phuyal et al. (2020) IC_50_ value is the concentration of the sample required to scavenge 50% of DPPH free radical. The IC₅₀ of pineapple extract represents the concentration required for 50% radical scavenging activity and expresses the extract's antioxidant potency.

(3) $$\%\mathrm{RSA}=\frac{\;\mathrm{AC}–\mathrm{AS}}{\mathrm{AC}}\times 100\%$$

where % RSA = percent of radical scavenging ability, AC = absorbance of the control (1 mL ethanol + 1 mL DPPH solution), AS = absorbance of the sample solution.

##### 2.4.2.7 ABTS Radical Scavenging Activity

Similarly the ABTS radical scavenging activity of the extract was performed for the Scavenging ability on ABTS radicals and was evaluated in the procedure described by (Liu et al. 2018). 7 mM ABTS and 2.4 mM potassium persulfate solution (1:1 v/v) was prepared and incubated in the dark for 12 h to generate ABTS^+^ radicals. The solution was diluted with ethanol to an absorbance of 0.72 at 734 nm. Stock solutions were serially diluted (20–100 μg/mL), sealed with aluminum foil, and incubated in the dark for 30 min. Absorbance was measured at 734 nm using a UV–Vis spectrophotometer (JASCO V‐770) calibrated with ethanol. Free radical scavenging was calculated using Equation (4), with all samples analyzed in triplicate and results expressed as IC₅₀ values.

(4) $$\%\text{ABTS scavenging ability}=\frac{\;\mathrm{AC}–\mathrm{AS}}{\mathrm{AC}}\times 100\%$$

where % ABTS scavenging ability = percent of ABTS scavenging ability, AC = absorbance of the control (1 mL ethanol + 1 mL ABTS solution), AS, absorbance of the sample solution.

#### 2.4.3 Determination of Proximate Composition of Chickpea Milk and Cheese

Proximate composition, namely moisture content, crude protein, crude fat, crude fiber, and total ash of chickpea milk and cheese were determined by following the standard procedure of AOAC (2016). In addition, the carbohydrate content was determined by difference.

#### 2.4.4 Physicochemical and Functional Properties of Chickpea Milk

##### 2.4.4.1 pH

The pH of milk was measured with a calibrated digital pH meter (HI98194). After adjusting the milk to room temperature in a beaker, the electrode was immersed, and the reading was recorded once stabilized.

##### 2.4.4.2 Specific Gravity

Specific gravity of chickpea milk was measured using an OMEISHIBIZHONGJI 0–70 hydrometer. The milk was poured into a clean 100 mL cylinder, the hydrometer gently immersed, and the reading taken at the milk surface intersection with the scale.

##### 2.4.4.3 Titratable Acidity

Titratable acidity of milk samples was determined per AOAC (2005) using Equation (5). A 10 mL milk sample (*V*_s_) was measured for analysis. Phenolphthalein indicator was added (two to three drops of) to the milk sample and this was titrated against sodium hydroxide solution (Toffanin et al. 2015). The Acidity was expressed as percent lactic acid.

(5) $$\text{Acidity}\%=\frac{V_{t}\times 0.009\times 100}{V_{s}\times D}$$

where *V*_t_ = volume of 0.1 N sodium hydroxide solution, *V*_s_ = volume of milk sample, *D* = density of milk sample.

##### 2.4.4.4 Total Soluble Solids

TSS in chickpea milk was measured using a refractometer following AOAC (2016). The device was calibrated with distilled water, then a drop of milk was placed on the prism, and the Brix percentage reading was recorded.

##### 2.4.4.5 Viscosity

Viscosity of chickpea milk was measured using a Brookfield Viscometer (DV‐E, USA). The milk, kept at room temperature in a water bath, was transferred to a clean beaker, and the probe was immersed for measurement.

### 2.5 Characterization of Chickpea Cheese

#### 2.5.1 Yield of the Cheese

The cheese yield was determined according to (Salinas‐Valdés et al. 2015) using Equation (6).

(6) $$\text{Yield}\%=\frac{\text{weight of produced cheese}}{\text{weight of milk used}}\times 100$$

#### 2.5.2 Physical and Functional Properties of Cheeses

##### 2.5.2.1 Texture Analysis

Texture Profile Analysis (TPA) of chickpea cheese was performed using a texture analyzer. Samples were molded into 10 mm × 20 mm cylinders, kept at room temperature for 6 h so that the structural stability is attained, then stored at 4°C in order to maintain microbial stability and preserve structural integrity until testing. A 30 mm flat probe applied a 0.5 kg load at 1 mm/s to a 20 mm target. Hardness (peak force in first compression), cohesiveness (internal bonds making up the body), and adhesiveness (force required to remove the food from the palate during eating) were measured for quality assessment.

##### 2.5.2.2 Color Analysis

The color of chickpea cheese was measured using a colorimeter, recording L* (lightness: 0–100), a* (red‐green), and b* (yellow‐blue) values. Analyses were performed in triplicate.

##### 2.5.2.3 pH

The pH of cheese samples was measured using a calibrated digital pH meter (HI98194). About 10 g of cheese was mixed with distilled water (1:1 w/v) for 1–2 min to form a uniform slurry. The electrode was inserted into the slurry, and the reading was taken after stabilization. The electrode was rinsed between measurements, and the process was repeated in triplicate.

#### 2.5.3 Morphological and Molecular Analysis of Cheeses

##### 2.5.3.1 Scanning Electron Microscope (SEM) Analysis

Cheese samples were oven‐dried for 30 min to remove moisture, then gold‐coated using an ion sputter coater (POLARON SC7620). SEM imaging was conducted at an accelerating voltage of 10 kV.

##### 2.5.3.2 Fourier‐Transform Infrared (FTIR) Analysis

The cheese samples were analyzed for Fourier‐transform infrared spectroscopy (FTIR) following the process described by (Betances‐Salcedo et al. 2017). Cheese samples were frozen at 4°C, freeze‐dried, ground into powder, and pressed into pellets. Spectra were acquired in the 400–4000 cm^−1^ range at 1 cm^−1^ resolution.

#### 2.5.4 Microbiological Contents and Nutritional Composition of the Cheeses

##### 2.5.4.1 Microbiological Analysis

The microbial load of cheese samples was assessed using the viable plate count method. Nutrient agar (28 g/L) was autoclaved at 121°C for 15 min. One gram of cheese was dissolved in distilled water, followed by serial dilution. Prepared plates were incubated at 37°C for 24 h, and colonies were counted as CFU/g.

##### 2.5.4.2 Mineral Composition

Mineral content in chickpea milk was determined using Atomic Absorption Spectroscopy (AAS) after nitric acid digestion to break down the organic matrix. Mean concentrations were calculated and expressed in mg/kg.

#### 2.5.5 Sensory Properties of Cheeses

Sensory evaluation of optimized and control cheese (appearance, aroma, texture, taste, and overall acceptability) was conducted by 30 untrained panelists (20 females, 10 males, aged 23–35), primarily university students. Testing took place in a sensory lab under controlled lighting and ventilation. Cheese cubes coded with 3‐digit numbers were served in randomized order. A 5‐point hedonic scale (1 = dislike extremely, 5 = like extremely) was used. Panelists rinsed with tap water between samples and were guided on the procedure. Appearance referred to visual traits (e.g., color, uniformity), aroma to olfactory stimuli, and texture to perceived softness. The cheese taste is a complex sensation comprising aroma, taste, and texture (Magalhães et al. 2011). The overall acceptability is the acceptance of panelists.

### 2.6 Data Analysis and Software

Design Expert (version) was used for experimental design and statistical analysis. Results were reported as mean ± SD (*n* = 3). ANOVA and Tukey's test (*p* < 0.05) were performed using Minitab to determine significant differences. Model validity was assessed using ANOVA, *R* ^2^, and adjusted *R* ^2^ values.

## 3 Result and Discussion

### 3.1 Proximate Composition of Chickpea Milk

The moisture content of milk is 78.42% ± 0.04% (Table 3). According to Shakeel et al. (2015) whenever the moisture content is decreased, the other nutritional components in the milk are enhanced.

**TABLE 3:** Proximate analysis of chickpea milk.

Composition of Chickpea milk					
Moisture (%)	Protein (%)	Fat (%)	Fiber (%)	Ash (%)	Carbohydrate (%)
78.42 ± 0.04	3.02 ± 0.01	3.5 ± 0.03	0.005 ± 0.003	0.35 ± 0.01	14.71 ± 0.07

*Note:* All values are presented as mean ± SD (triplicate analysis).

The protein content of chickpea milk is 3.02% ± 0.01% which is higher compared the studies as provided in (Table 3). The fat content was 3.5% ± 0.03%. The fat content agrees with the result reported by (Kishor et al. 2017) which is ranged from 3.4%–8.8% for Kabuli type. However, it showed an increment from the result 0.39%–0.5% reported by Duarte et al. (2022). The fat content of milk influences cheese texture and enhances flavor, while also providing essential fatty acids. However; the result of this study is not in agreement with (Hamioud 2025) report as chickpea milk is categorized as a fat‐free alternative (containing 0.39%–0.5% g/100 g) and is particularly comparable to oat and rice milk, which also contain similarly low levels of fat (0.1 and 0.3 g/100 mg, respectively) when compared to other milk substitutes. The fiber content in processed chickpea milk was low (0.005% ± 0.003%), likely due to removal during straining, as fiber mainly comes from residual pulp.

Ash content was 0.35% ± 0.01%, higher than Duarte's reported range (0.16%–0.62%). Total carbohydrates were 14.71% ± 0.07%, reflecting moderate levels compared to other legume milks like rice and peanut. This may contribute to the milk coagulation by lowering the pH, as higher carbohydrates, particularly sugars, can slightly lower the pH of the milk (Vénica et al. 2018).

### 3.2 Yield of Chickpea Milk and Pineapple Extract

The yield of chickpea milk was 78.4% ± 0.1% while slurry to water ratio was 1:4. This figure is a little lower than the result obtained by (Rincon et al. 2020); this variation occurred probably due to variation in water to slurry ratio.

The percentage yield of pineapple extract was recorded as 64.8% ± 0.8%; which is in agreement with (Shaik and Chakraborty 2022); it is indicated that ultrasonic treatment increased the yield of pineapple liquid extract by 18%–22% compared to the untreated one.

### 3.3 Physicochemical and Functional Properties of Chickpea Milk

The pH, specific gravity, and acidity were 6.6 ± 0.3, 1.1 ± 0.06, and 0.1% ± 0.06% respectively (Table 4). According to Arise et al. (2020) with higher pH values correlating to increased almond milk substitution, potentially affecting the texture and overall quality of the soy cheese. (Oyeniyi et al. 2014) reported that the specific gravity of pure milk should range between 0.9396 and 1.5125. If it is a little below 0.9396, it is still regarded as pure, but if it is higher than 1.5125, it is impure. The acidity of chickpea milk was lowered probably due to an increment of its pH. According to Yadav et al. (2016) report the acidity of soya milk increased with a lowering of the pH.

**TABLE 4:** Physicochemical and functional properties of chickpea milk.

Sample	Parameters	Values
Chickpea milk	pH	6.6 ± 0.3
	Specific gravity	1.1 ± 0.06
	Titratable acidity %	0.1 ± 0.06
	TSS (°Bx)	2.4 ± 0.05
	Viscosity (cP)	9.07 ± 0.03

*Note:* All the values are means ± standard deviations.

Total soluble solid and viscosity were 2.4 ± 0.05 ^0^Bx and 9.07 ± 0.03 respectively. The TSS in chickpea milk can lead to better curd formation by enhancing the emulsifying capacity of the milk (Guemra et al. 2024). Overlay high viscosity impairs flow ability, while low viscosity may promote sedimentation and flocculation of colloidal particles (Grossmann et al. 2021). The viscosity of chickpea milk is a little lower than the result reported by (Kale et al. 2012); which was a study on soya milk. This is probably due to their fat content; literature indicated that as the fat content in milk increases the milk generally becomes thicker.

#### 3.3.1 Physicochemical and Functional Properties of Pineapple Extract

##### 3.3.1.1 pH and Total Soluble Solids Analysis

The pH and total soluble solids of this study were recorded as 4.02 ± 0.01 and 12.01 ± 0.01 (°Brix) respectively; the data were expressed as means ± SD. The total soluble solids content expressed as ^0^Brix and the pH of the pineapple liquid extract analyzed in this study was compared to those in the literature (Das 2023) reports ranged from 14.71 ± 0.002–15.10 ± 0.005 (°Brix) and pH (3.7 ± 0.001–4.06 ± 0.005), respectively.

##### 3.3.1.2 Milk Clotting Activity (MCA)

The results obtained from the determination of MCA (U/mL) of the plant extracts are 50.18 ± 0.3 for pineapple, 28.6 ± 0.03 for pawpaw leaf, and 26.72 ± 0.09 for nettle leaf, respectively (see Table 5). Based on the ANOVA analysis, each treatment group exhibited a statistically significant difference (*p* < 0.05) in MCA. The highest activity was observed at the shortest clotting time (478 ± 2.9 s).

**TABLE 5:** MCA of plant extracts.

Sample coded	Milk clotting activity (U/mL)
A	50.18 ± 0.3^a^
B	28.6 ± 0.03^b^
C	26.72 ± 0.09^c^

*Note:* All the values are means ± standard deviations. Means in the same column with different superscripts are significantly different (*p* < 0.05).

As it is indicated in Table 5 the lowest coagulation time found in sample A with pineapple, followed by sample B with pawpaw leaf and sample C with nettle leaf.

**FIGURE 4:**
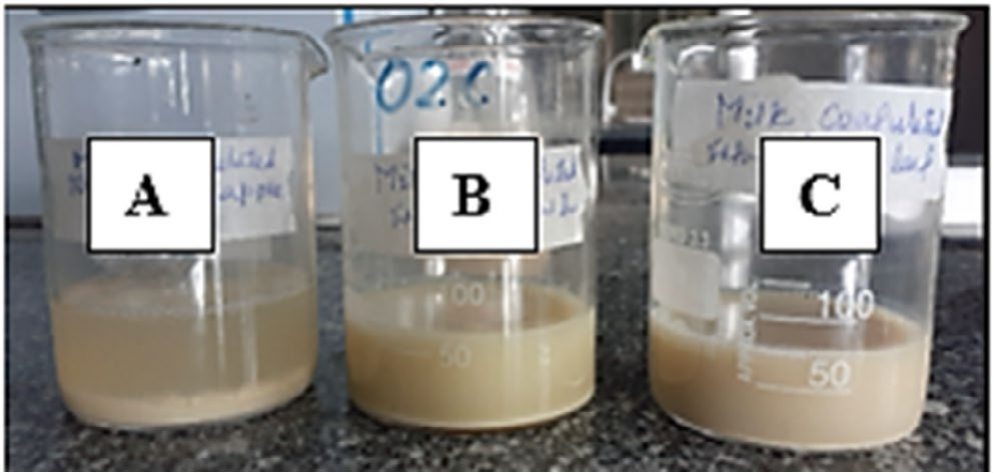
MCA test of Pine Apple (A), Papaw leaf (B) and Nettle leaf (C) Extracts.

As illustrated in Figure 4, the observation is done when the milk clots with slight yellowish liquid appearance indicating the separation of the insoluble from the soluble protein particles. Similarly (Kartawiria et al. 2019) reported that shorter coagulation time was achieved with pineapple liquid extract to other coagulants in the preparation of soft cheese.

##### 3.3.1.3 Antimicrobial Activity

The antibacterial activity of pineapple extract was tested against *Staphylococcus aureus* (Gram‐positive pathogen causing various infections) and *Escherichia coli* (Gram‐negative bacterium linked to intestinal, urinary, and gastrointestinal infections) as provided below (Table 6).

**TABLE 6:** Antimicrobial activity of pineapple extract.

Sample	Tested bacteria	Zone of inhibition (mm)
Pine apple extract	*Staphylococcus aureus*	13.99 ± 0.02
	*Escherichia coli*	6.45 ± 0.05

*Note:* All the values are means ± standard deviations.

After 24 h, pineapple extract inhibited both *S. aureus* and *E. coli* , with zones of inhibition of 13.99 ± 0.02 and 6.45 ± 0.05 mm, respectively. The result for *Staphylococcus aureus* is in agreement with (Ogunmefun et al. 2018) in which the pineapple juice extract showed highest zone of inhibition for *S. aureus* as 16 mm. While the result for *Escherichia coli* is not in agreement with the above study in which no zone of inhibition was observed.

##### 3.3.1.4 Antioxidant Activity

In this study, the antioxidant potential of the extract was evaluated using DPPH and ABTS methods. Curve showing the relation among extract concentration and its inhibition percentage (%RSA) have been developed in comparison with ascorbic acid (Figures 11 and 12). The radical scavenging activity was calculated from the dose response curve and expressed as IC50 (extract concentration that neutralizes 50% of free radicals). IC50 is the concentration at which the antioxidant brings about 50% scavenging of the radical (Kanatt et al. 2018).

**FIGURE 5:**
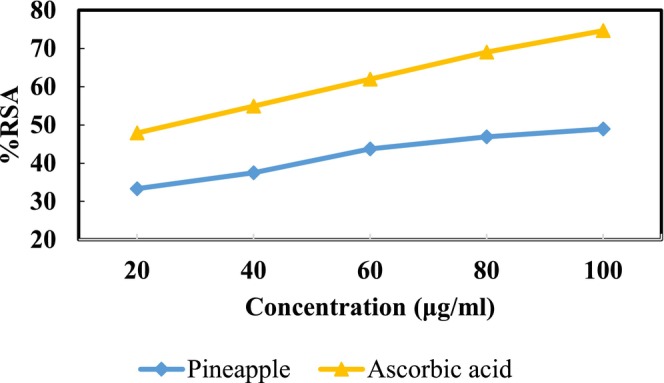
DPPH profiles of pineapple extract against ascorbic acid.

**FIGURE 6:**
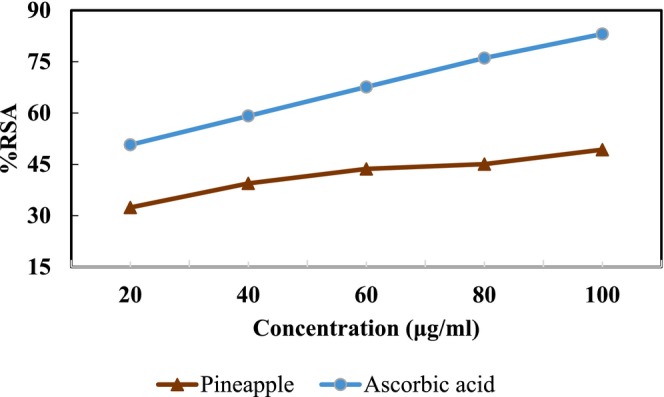
ABTS profiles of pineapple extract against ascorbic acid.

The applied methods revealed that pineapple extract possessed excellent antioxidant activity, which is in agreement with (Yuris and Siow 2014). The radical scavenging ability (RSA) of pineapple extract ranged from (33.33%–48.96%) in the DPPH method (Figure 5 above) with an IC50 value of 233.021 μg/mL and in the ABTS method % RSA ranged from (32.39%–49.29%) (Figure 6 below) with 227.33 μg/mL of IC50 μg/mL. Similar findings were reported by (Putri et al. 2018).

Ascorbic acid could probably be the main contributor toward the radical scavenging activity of the pineapple extract as ascorbic acid is a free radical scavenger which has a very fast reaction compared to other radical scavenging molecules such as the phenolic compounds (Scalzo 2008). Additionally, a study on antioxidant activity of pineapple peel revealed that all pineapple extracts including water and ethanol extracts were quite effective in scavenging DPPH− and ABTS+—radicals and reducing power (Putri et al. 2018).

### 3.4 Response Surface Methodology (RSM)

The effects of MER (A), CT (B), and Ct (C) on yield (Y %), texture (Ha Co, Ad), color (L*, a*, b*), and pH were evaluated through 17 randomized runs. Table 7 below presents the results for each response based on different parameter combinations.

**TABLE 7:** Box–Behnken design employed for formulation of chickpea cheese.

Runs	MER	CT (°C)	Ct (min)	Factors				Responses			
				Y (%)	Ha (N)	Co (N)	Ad (N)	L*	a*	b*	pH
1	0.3	50	60	36.16	40.18	0.599	1.93	76.37	1.91	32.79	5.65
2	0.1	60	45	21.41	29.22	0.33	1.71	81.25	1.29	28.29	6.33
3	0.2	50	45	31.02	34.96	0.41	2.13	79.48	1.69	30.24	5.69
4	0.1	50	30	20.05	25.01	0.37	1.73	81.42	1.32	27.89	6.47
5	0.2	40	30	23.27	25.58	0.32	1.9	82.58	1.21	28.28	5.92
6	0.3	50	30	28.06	34.29	0.46	1.99	78.84	1.73	31.27	5.27
7	0.2	40	60	24.23	32.98	0.56	2.07	83.05	1.82	29.02	5.77
8	0.2	50	45	31.01	34.96	0.41	2.13	79.48	1.7	30.25	5.71
9	0.2	50	45	31.04	34.94	0.41	2.14	79.47	1.69	30.26	5.69
10	0.1	40	45	28.77	24.22	0.35	1.72	82.29	1.23	27.36	6.23
11	0.3	40	45	35.86	35.11	0.51	1.89	79.25	1.71	31.12	5.61
12	0.2	50	45	31.01	34.96	0.41	2.13	79.46	1.68	30.27	5.68
13	0.2	60	60	25.02	34.88	0.37	1.91	79.53	1.37	30.36	5.82
14	0.2	60	30	19.29	33.41	0.52	1.94	82.47	1.83	29.53	5.72
15	0.2	50	45	30.99	34.96	0.412	2.12	79.45	1.71	30.24	5.67
16	0.1	50	60	18.61	27.97	0.32	1.95	81.41	1.31	27.92	6.01
17	0.3	60	45	40.02	39.82	0.541	1.77	76.67	1.82	32.78	5.37

#### 3.4.1 Model Equation

The following equations illustrate the effects of linear, quadratic, and interaction terms on cheese properties.

(7) $$\begin{array}{c} Y\%=31.01+6.41A-0.7987B+1.67C+2.88\mathrm{AB}\\ \;+2.38\mathrm{AC}+1.19\mathrm{BC}+1.63A^{2}-1.13B^{2}-6.93C^{2} \end{array}$$

(8) $$\begin{array}{c} \mathrm{Ha}\;\left(N\right)=34.96+5.37A+2.43B+2.21C-0.725\mathrm{AB}\\ \;+0.7325\mathrm{AC}-1.48\mathrm{BC}-1.36A^{2}-1.51B^{2}-1.74C^{2} \end{array}$$

(9) $$\begin{array}{c} \mathrm{Co}\left(N\right)=0.4104+0.092A+0.0024B+0.0225C+0.0132\mathrm{AB}\\ \;+0.0469\mathrm{AC}+0.0975\mathrm{BC}+0.086A^{2}+0.042B^{2}+0.0179C^{2} \end{array}$$

(10) $$\begin{array}{c} \mathrm{AD}\left(N\right)=2.13+0.0588A-0.0312B+0.0375C-0.0275\mathrm{AB}\\ \;-0.0700\mathrm{AC}-0.0500\mathrm{BC}-2062A^{2}-0.1513B^{2}-0.0237C^{2} \end{array}$$

(11) $$\begin{array}{c} L*=79.47-1.91A-0.9063B-0.6188C-0.3850\mathrm{AB}\\ \;-0.61.50C-0.8525\mathrm{BC}-1.00A^{2}+1.40B^{2}+1.04C^{2} \end{array}$$

(12) $$\begin{array}{c} b*=30.35+2.06A+0.6475B+0.3900C+0.1825\mathrm{AB}\\ \;+0.3725\mathrm{AC}+0.0225\mathrm{BC}+0.1527A^{2}-0.5172B^{2}-0.4373C^{2} \end{array}$$

(13) $$\begin{array}{c} a*=1.69+0.2525A+0.0425B+0.0400C+0.0125\mathrm{AB}\\ \;+0.0475\mathrm{AC}-0.2675\mathrm{BC}-0.0858A^{2}-0.0957B^{2}-0.0408C^{2} \end{array}$$

(14) $$\begin{array}{c} \mathrm{pH}=5.69-0.3925A-0.0363B-0.0163C-0.0850\mathrm{AB}\\ \;+0.2100\mathrm{AC}+0.0625\mathrm{BC}+0.1197A^{2}+0.0773B^{2}+0.0422C^{2} \end{array}$$

#### 3.4.2 Fit Statistics

The model fit was confirmed by the close agreement between predicted and adjusted *R* ^2^ values (Table 8) below.

**TABLE 8:** Tables of ANOVA for the model fitting.

Parameters	Cheese properties							
	Y %	Ha (N)	Co (N)	Ad (N)	L*	b*	a*	pH
*R* ^2^	1.0000	1.0000	1.0000	0.9993	1.0000	0.9994	1.0000	0.9993
Adjusted *R* ^2^	1.0000	1.0000	0.9999	0.9983	1.0000	0.9986	1.0000	0.9985
Predicted *R* ^2^	0.9999	1.0000	0.9999	0.9960	1.0000	0.9982	1.0000	0.9973
Adeq precision	1183.2750	2687.3099	520.7750	87.3688	867.3842	101.1426	693.6043	127.9777

*Note:* The coefficient of variation (CV %) for all cheese properties was below 10%, indicating strong model reliability. Additionally, the close alignment of actual and predicted values along the straight line confirms good model fit.

#### 3.4.3 3D Surface Plot of Interaction Effect

##### 3.4.3.1 Interaction Effects of MER, CT and Ct on the Yield of Chickpea Cheese

**FIGURE 7:**
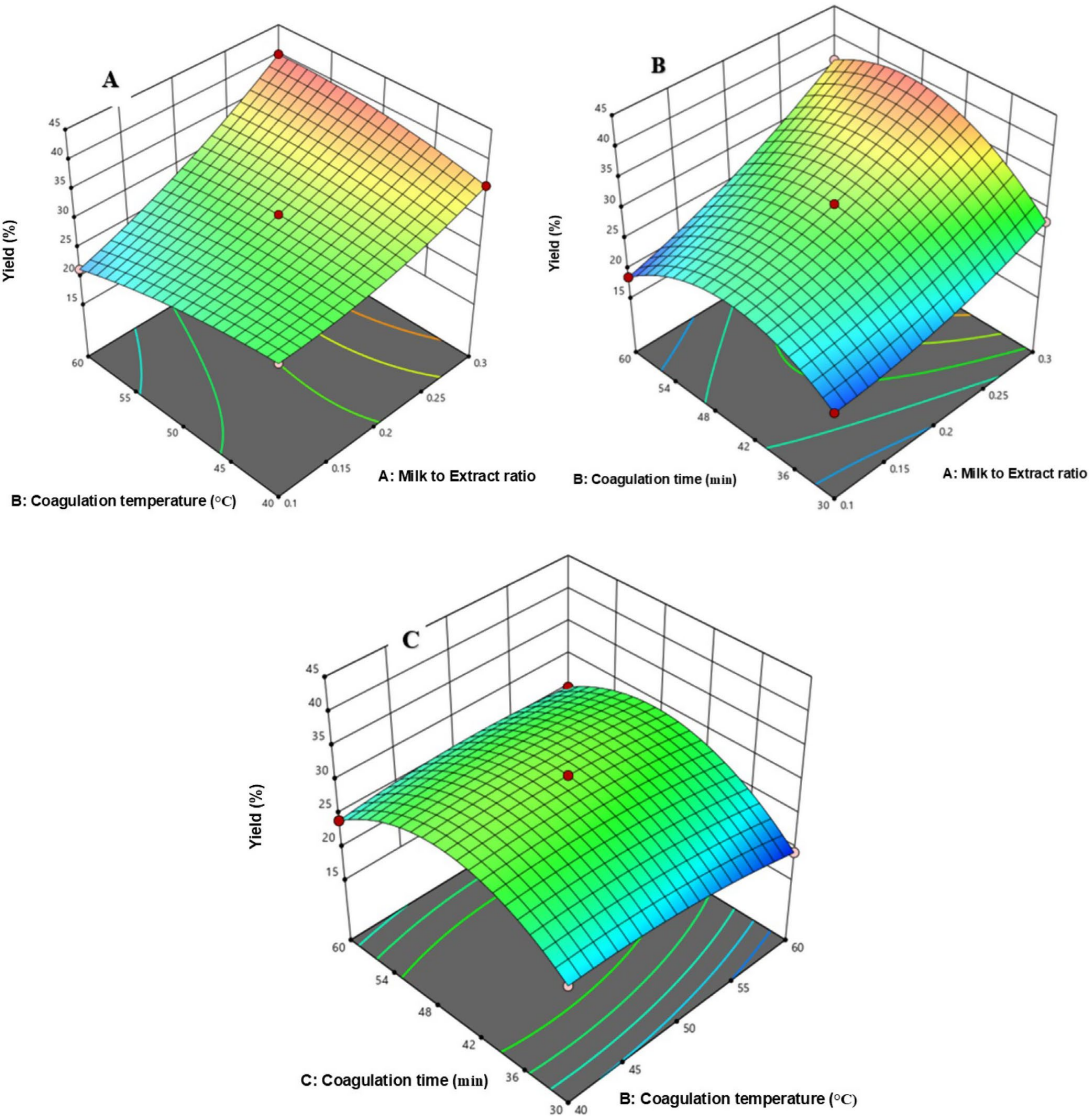
The response surface for yield of cheese as a function of CT and MER (A), Ct and MER (B), and CT and Ct (C).

In Figure 7A, yield increased with rising coagulation temperature (40°C–60°C) and milk‐to‐extract ratio (0.1–0.3). Figure 7B shows yield also rose with increased milk‐to‐extract ratio and coagulation time (30–60 min). Figure 7C demonstrates that higher temperature and longer coagulation time further enhanced yield.

##### 3.4.3.2 Interaction Effects of the Independent Variables on the Textural Properties of Chickpea Cheese

###### 3.4.3.2.1 Hardness

**FIGURE 8:**
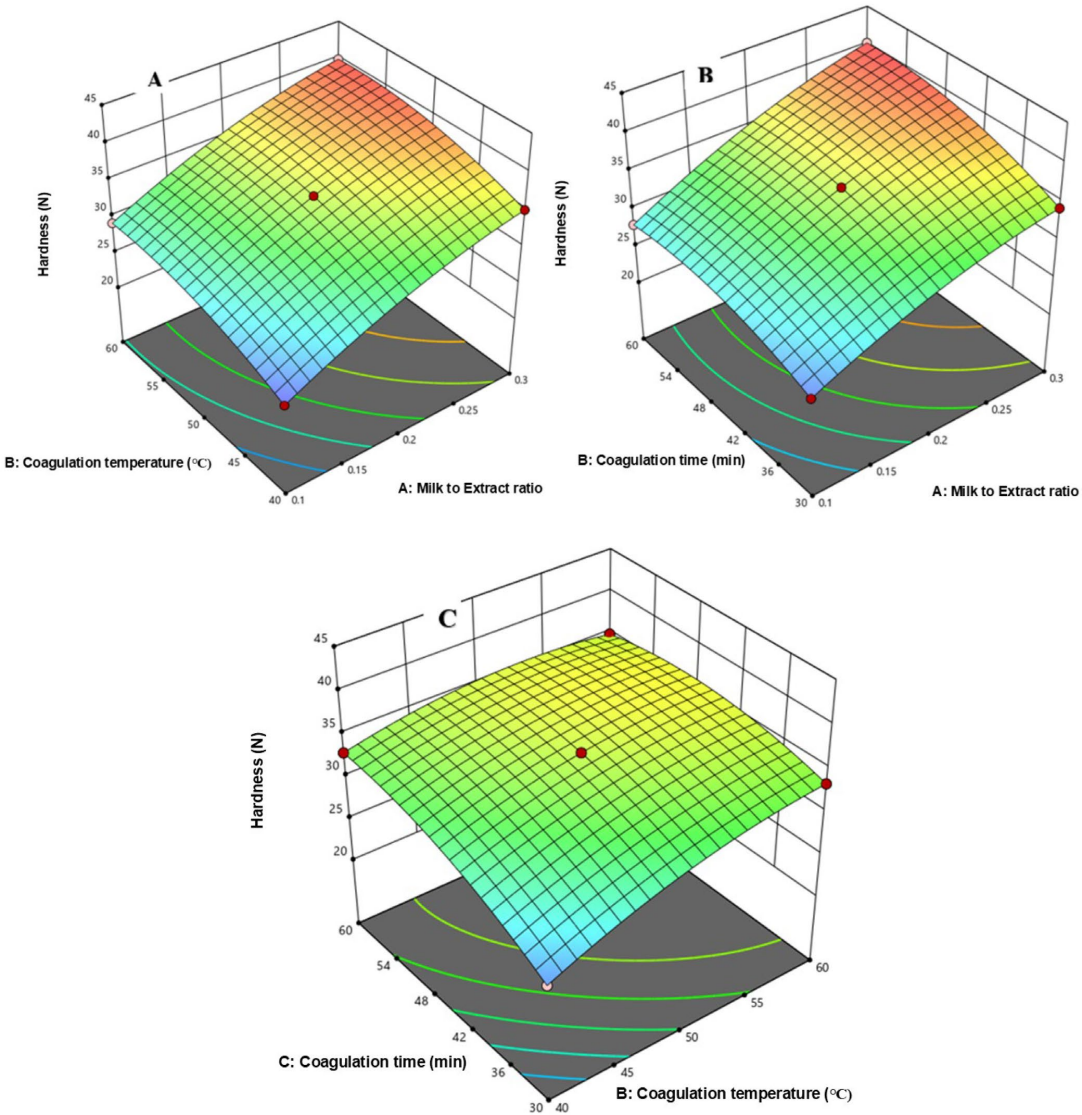
The response surface for cheese Hardness as a function of CT and MER (A), Ct and MER (B), and CT and Ct (C).

In Figure 8A, hardness increased with higher coagulation temperature (40°C–60°C) and milk‐to‐extract ratio (0.1–0.3), likely due to greater protein denaturation at elevated temperatures. Figure 8B shows increased hardness with longer coagulation time (30–60 min), and Figure 8C confirms that both higher temperature and extended time further enhanced hardness.

###### 3.4.3.2.2 Cohesiveness

**FIGURE 9:**
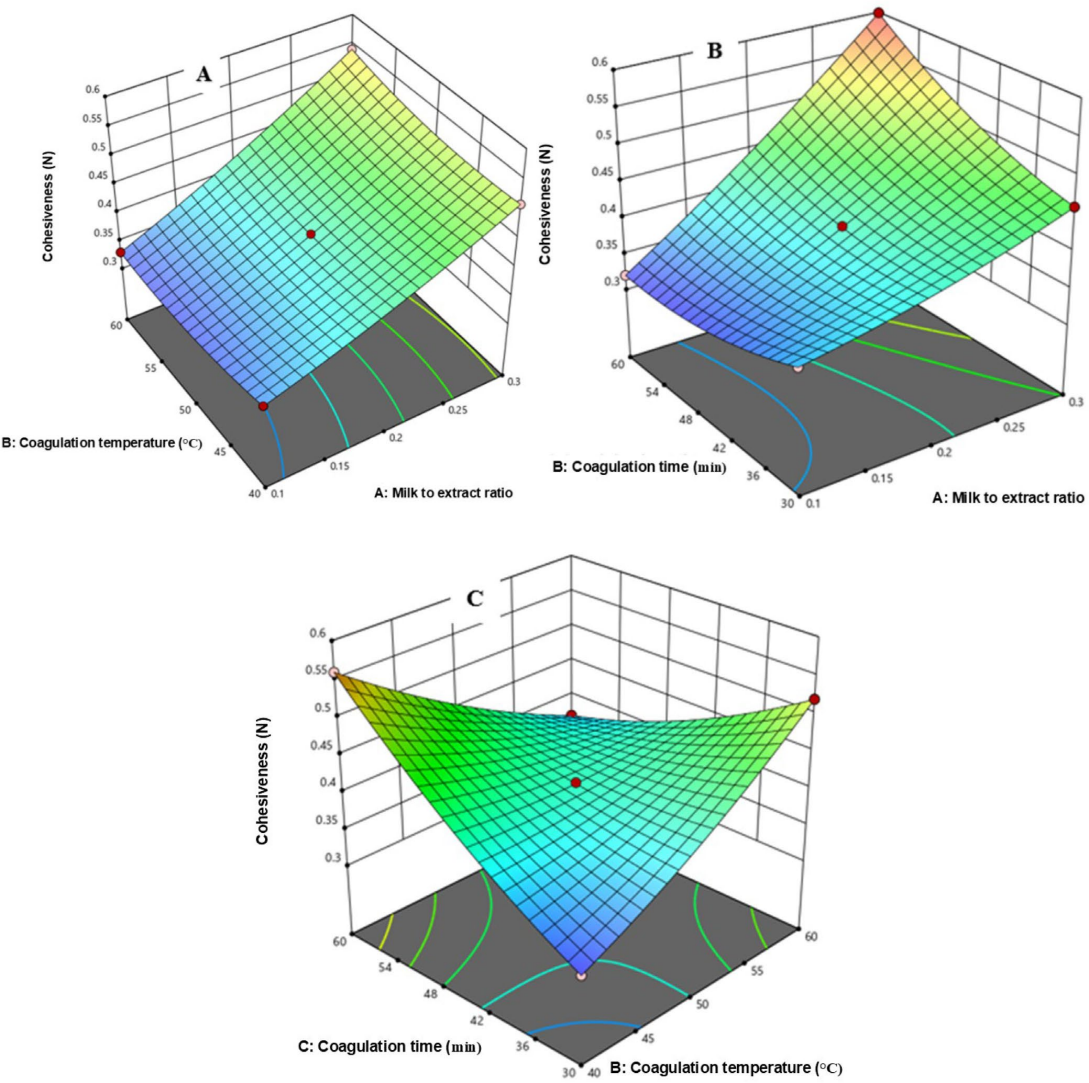
The response surface for cheese Cohesiveness as a function of CT and MER (A), Ct and MER (B), and CT and Ct (C).

Figure 9A demonstrates that cohesiveness of the cheese is increased with the increase of Milk to extract ratio from 0.1 to 0.3 while decreased with increasing of coagulation temperature from 40°C to 60°C. Figure 9B shows increased Cohesiveness with longer coagulation time (30–60 min) and increased Milk to extract ratio from 0.2 to 0.3, and Figure 9C shows rising of coagulation time decreased the cohesiveness of the cheese.

##### 3.4.3.3 Adhesiveness

**FIGURE 10:**
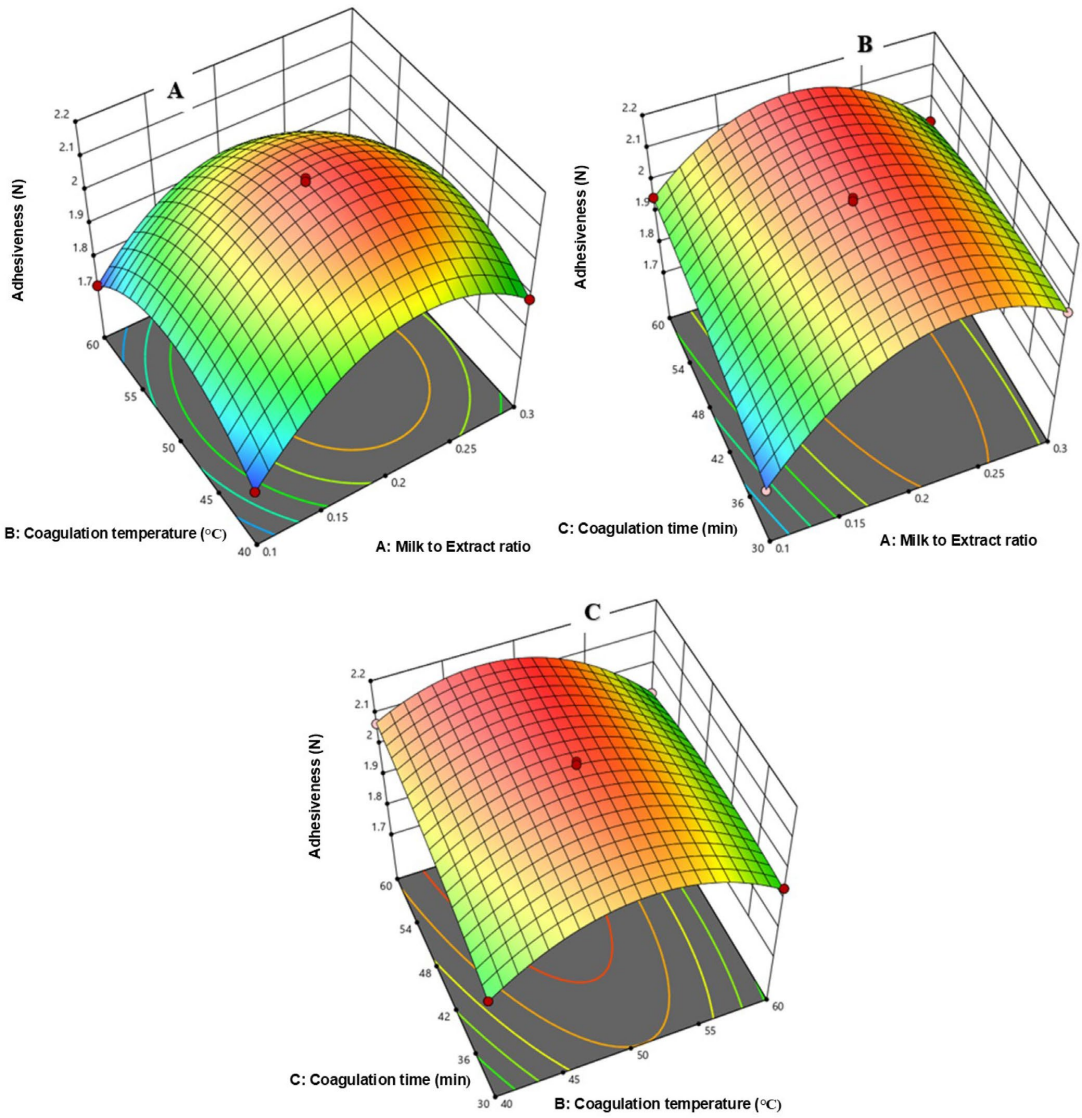
The response surface for cheese Adhesiveness as a function of CT and MER (A), Ct and MER (B), and CT and Ct (C).

In Figure 10A, adhesiveness increased with a higher milk‐to‐extract ratio but decreased with rising coagulation temperature. Figure 10B shows that a longer coagulation time (30–60 min) increased adhesiveness. In Figure 10C, increasing both temperature and time reduced adhesiveness, likely due to decreased moisture content.

##### 3.4.3.4 Interaction Effects of the Independent Variables on the Color Properties of Chickpea Cheese

**FIGURE 11:**
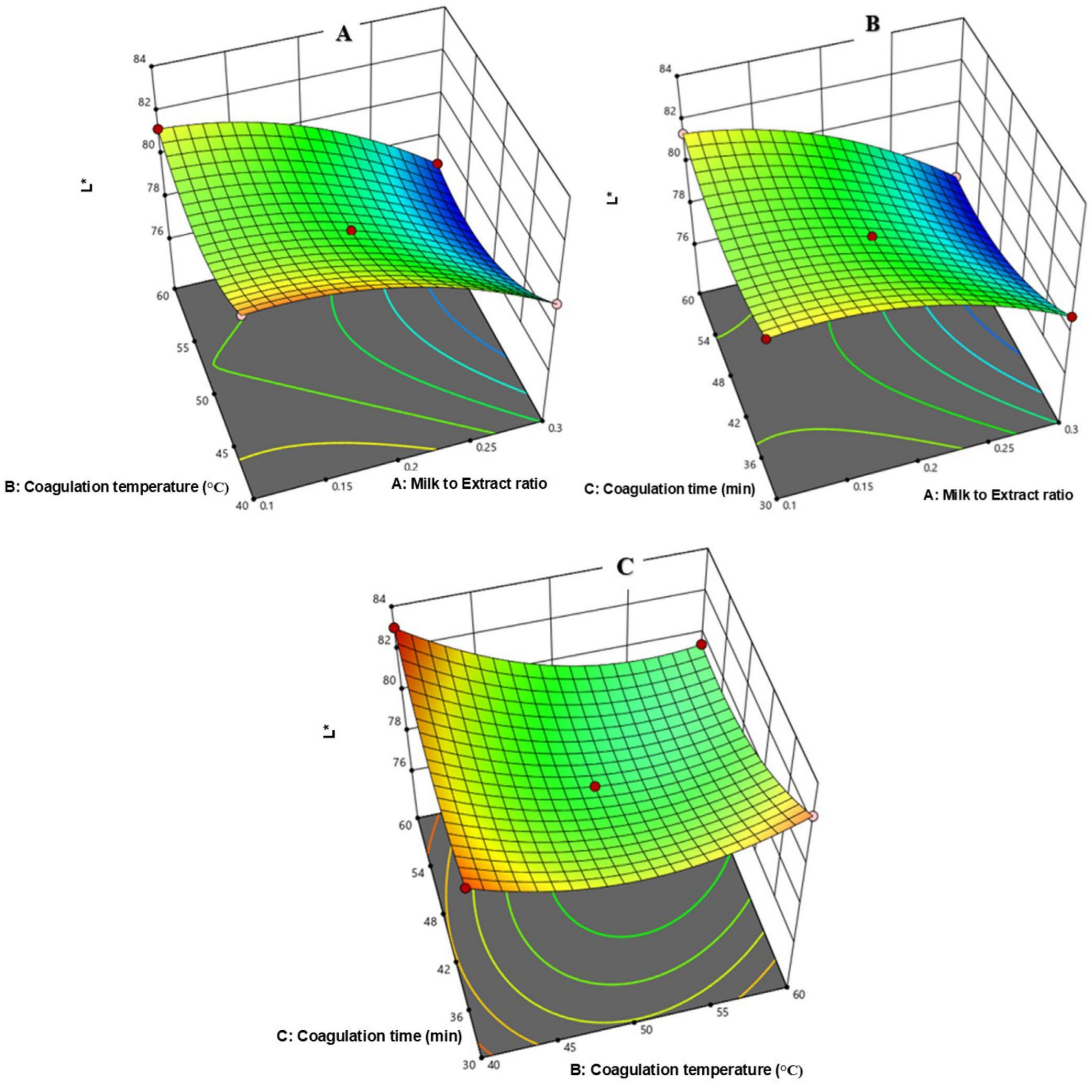
The response surface for cheese L* as a function of CT and MER (A), Ct and MER (B), and CT and Ct (C).

In Figure 11A, L* increased from 76.37 to 83.05 with lower milk‐to‐extract ratio (0.3 to 0.2) and temperature (40°C to 50°C). In Figure 11B, L* decreased from 78.84 to 76.37 as coagulation time increased (30–60 min). Figure 11C shows that increasing both temperature and time reduced L* from 82.58 to 79.48.

**FIGURE 12:**
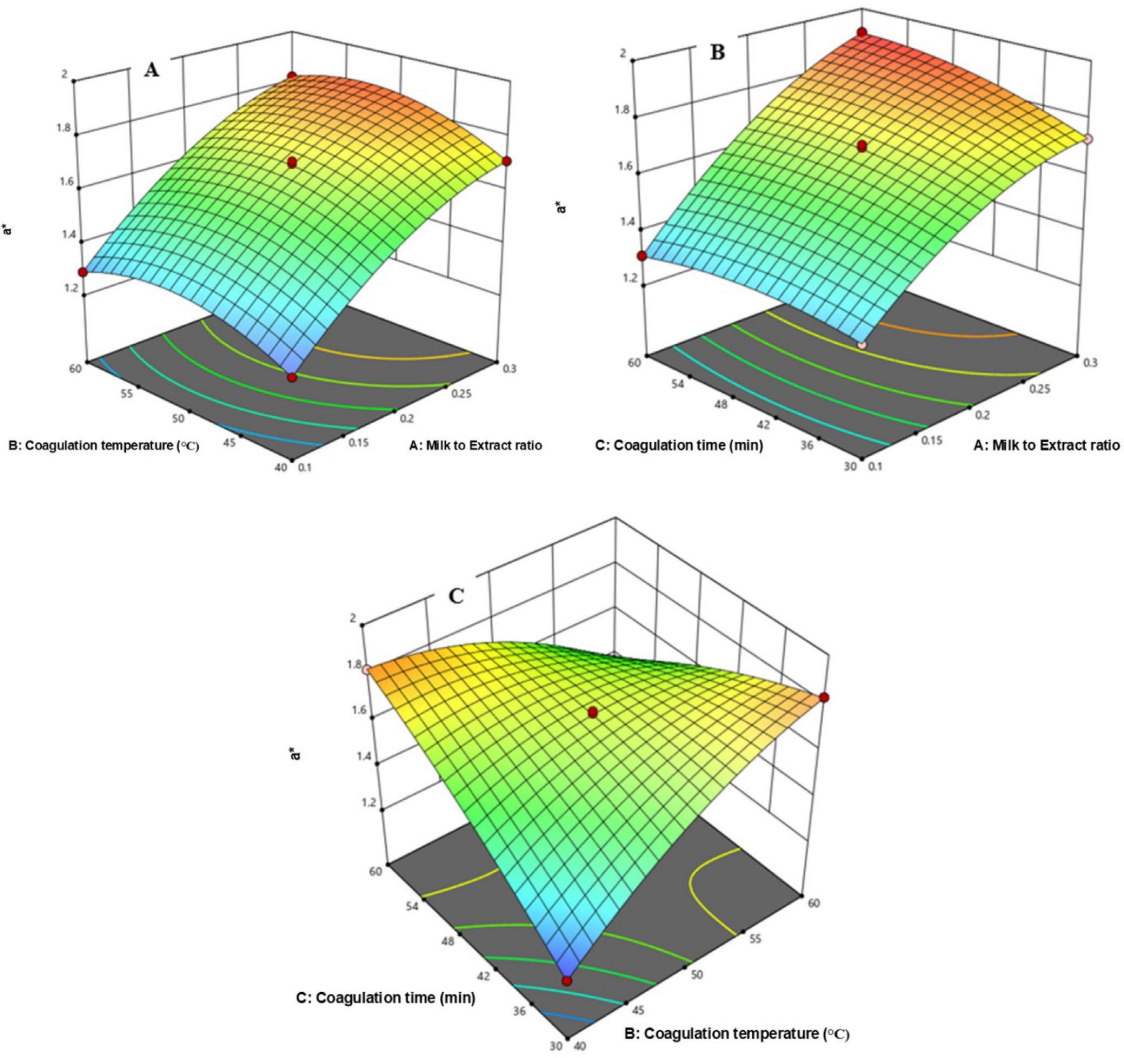
The response surface for a* of cheese as a function of CT and MER (A), Ct and MER (B), and CT and Ct (C).

Figure 12A illustrates the effects on a* (redness) value of chickpea cheese. In Figure 12A, a* increased from 1.82 to 1.91 with rising milk‐to‐extract ratio and coagulation temperature. Figure 12B shows a* rose from 1.69 to 1.91 with increasing MER (0.1 to 0.3) and coagulation time. In Figure 12C, a* increased from 1.71 to 1.91 with CT (40°C–50°C) and Ct (45–60 min), but decreased when Ct was lowered while CT rose to 60°C.

**FIGURE 13:**
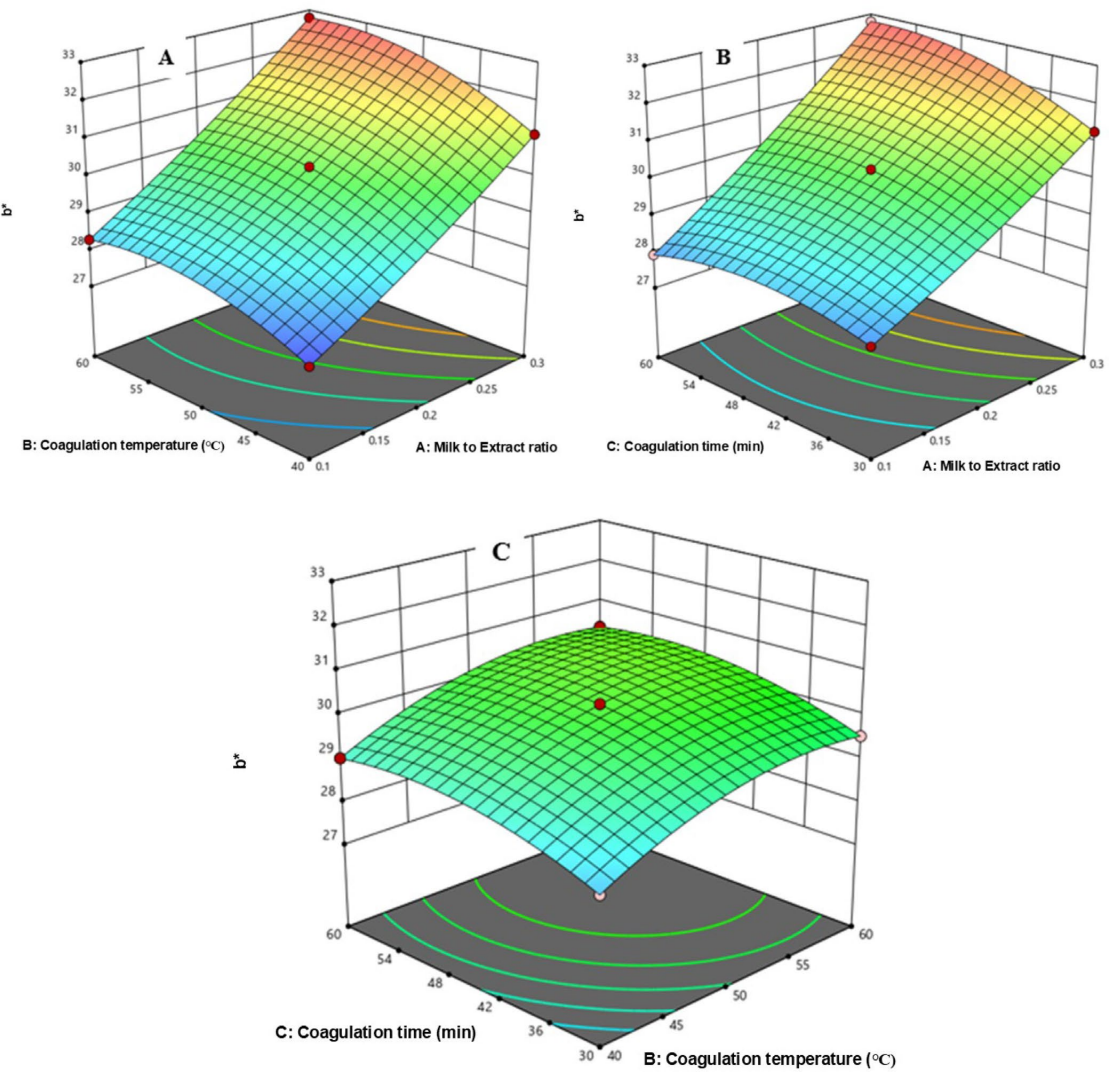
The response surface for b* of cheese as a function of CT and MER (A), Ct and MER (B), and CT and Ct (C).

In Figure 13A, b* increased with higher milk‐to‐extract ratio (0.1 to 0.2) and decreased coagulation temperature (60°C–50°C). b* also rose from 27.36 to 30.24 with simultaneous increases in both factors. Figure 13B shows b* decreased from 31.27 to 30.25 when milk‐to‐extract ratio dropped (0.3 to 0.2) at longer coagulation times, but increased with higher ratio and time. Figure 13C indicates yellowness rose with increasing temperature and coagulation time. Similarly, a pronounced yellowness was observed on cheese spread at higher coagulation temperature and extended coagulation time (Karimidastjerd et al. 2024).

##### 3.4.3.5 Interaction Effects of the Independent Variables on pH of Chickpea Cheese

**FIGURE 14:**
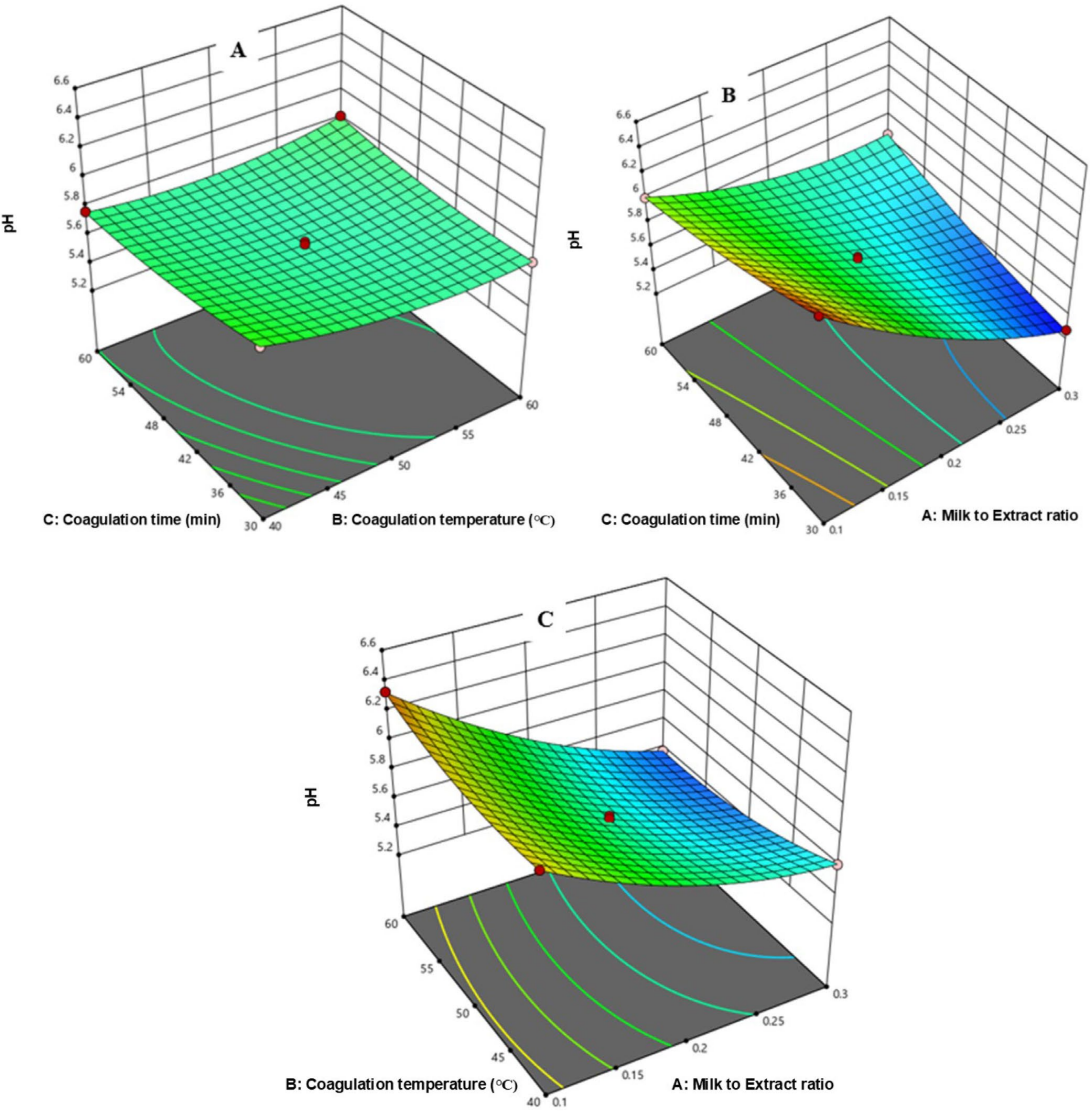
The response surface for pH of cheese as a function of CT and MER (A), Ct and MER (B), and CT and Ct (C).

In Figure 14A, pH decreased from 5.77 to 5.65 with a higher milk‐to‐extract ratio (0.2 to 0.3) and coagulation temperature (40°C–50°C). Figure 14B shows pH similarly declined with increasing milk‐to‐extract ratio and coagulation time. The same result was obtained by (Komansilan et al. 2023); as the extract from pineapple is added, the pH of cheese curds reduced. As he reported, the highest pH (6.8) is observed in the cheese sample without addition of the bromelain while the lowest pH (5.05) was observed in the cheese sample after addition of bromelain. However, the pH increased from 5.69 to 5.77 at extended coagulation time from 45 min to 60 min with reduced coagulation temperature Figure 14C.

#### 3.4.4 Optimization and Validation

A numerical response optimization technique was applied to determine the optimum combination of the factors. The optimum values were predicted by the response surface analysis of the combined variables. A trial cheese was developed by using the optimized formula to validate this estimation and triplicate tests were conducted. Percentage relative errors were used to determine the reliability of the model; it was calculated using Equation (15).

(15) $$\text{Relative error}\;\left(\%\right)=\left(\frac{\text{Predicted}-\text{Actual}\;}{\text{Predicted}}\right)\times 100\%$$

### 3.5 Characterization of Optimized and Control Chickpea Cheese

**FIGURE 15:**
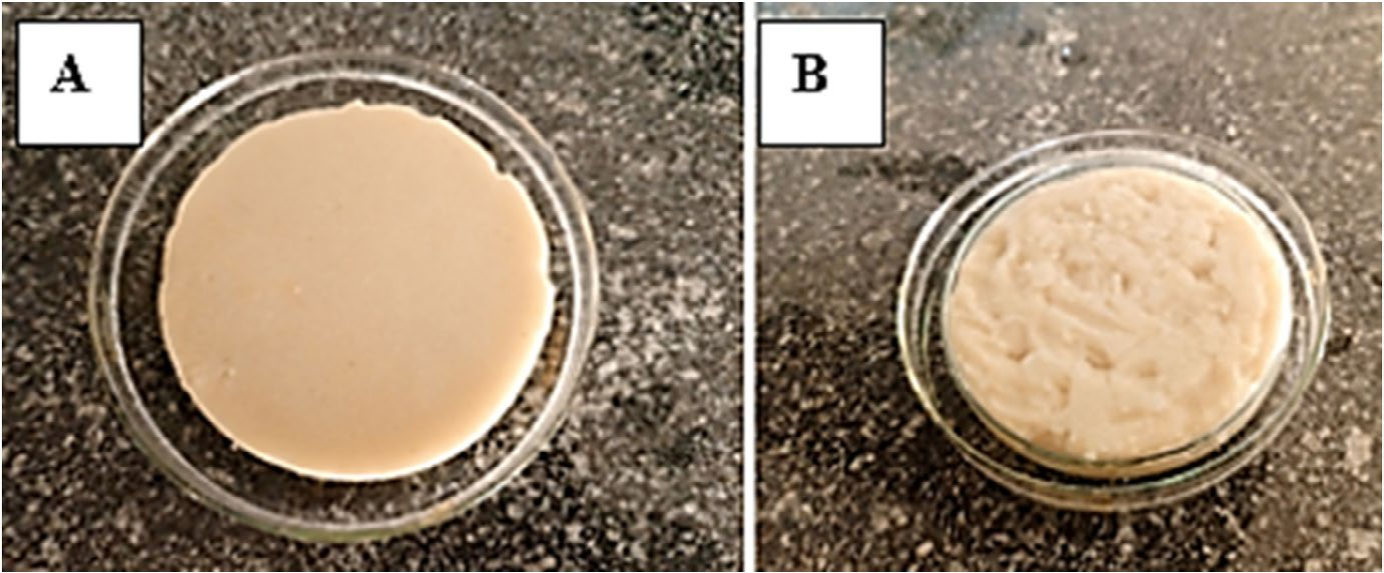
Developed chickpea‐based cheese samples: Cheese with pineapple extract (A) and cheese without pineapple extract (B).

Chickpea‐based cheese was prepared using pineapple extract (CP, optimized cheese) and without it (CC, control cheese) under similar conditions to ensure valid comparison. Visual representations are shown in Figure 15 below.

#### 3.5.1 Yield and Proximate Compositions of Chickpea Cheese Samples

As it is indicated in (Table 9) the moisture, protein, fat, fiber, ash and carbohydrate for CP 65.15% ± 0.12%, 9.77% ± 0.05%, 4.13% ± 0.01%, 0.51% ± 0.02%, 2.03% ± 0.01% and 18.4% ± 0.2%, respectively and for CC 67.3% ± 0.04%, 6.2% ± 0.03%, 4.09% ± 0.04%, 0.84% ± 0.01%, 1.94% ± 0.04% and 19.64% ± 0.1% was observed. Cheese coagulated with pineapple extract showed lower moisture and higher protein content compared to the control. This effect is probably due to bromelain‐mediated modification of milk proteins. Bromelain enzyme in pineapple partially hydrolyzes casein, exposing additional reactive sites that enhance interaction during coagulation. This promotes a compacted curd matrix with greater protein retention, while reducing the amount of free moisture trapped in the curd. However, the fat content is nearly identical in both cheese samples, which indicates that incorporation of pineapple extract does not significantly alter fat retention during cheese formation.

**TABLE 9:** Yield and proximate composition of chickpea cheeses.

Parameters %	Yield and proximate composition of chickpea cheese samples	
	CP	CC
Yield	27.54 ± 0.1^a^	18.89 ± 0.13^b^
Moisture	65.15 ± 0.12^b^	67.3 ± 0.04^a^
Protein	9.77 ± 0.05^a^	6.2 ± 0.03^b^
Fat	4.13 ± 0.01^a^	4.09 ± 0.04^a^
Fiber	0.51 ± 0.02^b^	0.84 ± 0.01^a^
Ash	2.03 ± 0.01^a^	1.94 ± 0.04^b^
Carbohydrate	18.4 ± 0.2^b^	19.64 ± 0.1^a^

*Note:* All the values are means ± standard deviations. Means within a row with different letters are significantly different at *p* < 0.05.

Abbreviations: CC, control cheese sample without pineapple extract; CP, optimized cheese sample with pineapple extract.

The chickpea cheese coagulated with pineapple extract showed lower fiber content than that of the control chickpea cheese. The lower fiber content in CP may reflect residual plant polysaccharides from the pineapple extract. The ash contents of the cheese samples were generally low. However, these values are in agreement with previous reports for cheese made from soya (Balogun et al. 2019). However, the slight increase in ash content in CP suggests that pineapple extract may contribute to mineral retention, possibly due to better coagulation and binding of minerals. The carbohydrate content showed slight variation between the samples, likely due to the breakdown of complex carbohydrates in to simpler sugars by pineapple extract.

Yield of cheese samples ranged from 18.89% ± 0.13% (CC; cheese developed without extract) to CP (27.54% ± 0.1%). The addition of pineapple extract initiated the curdling of available protein in the milk, and this may explain the improvement of the cheese yield. Related results were reported that the optimum coagulant concentration of pine apple extract can initiate efficient coagulation of the milk, resulting in a compact curd and higher resulting cheese (Komansilan et al. 2023).

#### 3.5.2 Physical and Functional Properties of Cheeses

##### 3.5.2.1 Textural Property Analysis

**FIGURE 16:**
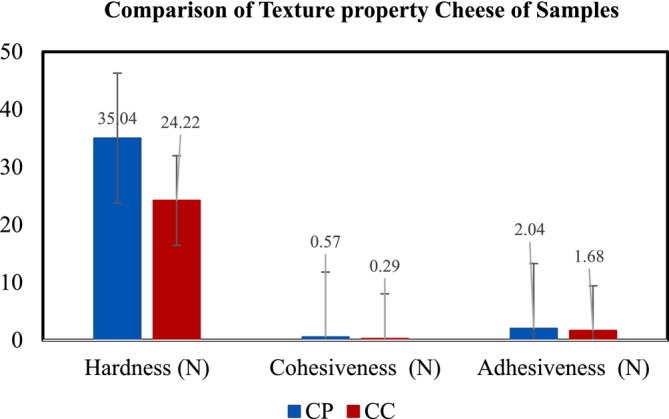
Comparison of textural properties: hardness, cohesiveness, and adhesiveness of chickpea cheeses (CP; optimized cheese and CC; control cheese).

According to Lamichhane et al. (2018) texture is often associated with the quality and identity of foods; regardless, consumer evaluation of texture occurs during mastication. As the results presented in (Figure 16) there was a significant difference (*p* < 0.05) between the optimized and the control cheese for the parameters specifically hardness and cohesiveness, which is probably due to enzymatic activity and chemical components in pineapple extract that lead to a denser protein network in optimized cheese.

Hardness is the force required to penetrate the sample or the force needed to attain a given deformation; cohesiveness is the amount of deformation undergone by a material before rupture when biting completely through the sample using the strength of the internal bonds making up the body of the product and adhesiveness is the force required to remove the food from the palate during eating (Uprit and Mishra 2004).

With respect to hardness and cohesiveness, the control cheese CC was softer and less compact than the optimized one CP, which shows the variation of protein density since the protein matrix is the structural component conferring greater resistance to deformation and resulted in reinforcing the gel structure and cohesiveness of the cheese (Cunha et al. 2010).

The optimized cheese CP also showed greater adhesiveness than the control cheese CC. The extent of the interaction between the fat and protein probably influenced the adherence between the product and the probe surface. Adhesiveness is related to the structure of the protein matrix and the interaction of fat and protein, which influences the adherence between the product and the contact surface (Grasso et al. 2022).

Incorporation of pineapple extract enhanced the textural property of chickpea cheese; hardness, cohesiveness, and adhesiveness for CP were 35.04 ± 0.01 N, 0.57 ± 0.01 N, and 2.14 ± 0.01 N, and CC 24.22 ± 0.01 N, 0.29 ± 0.01 N, 1.68 ± 0.01 N, respectively.

##### 3.5.2.2 Color Property Analysis

As illustrated in Table 10 the L*, b* and a* values for optimized cheese were 81.08 ± 0.02, 30.02 ± 0.01 and 1.89 ± 0.01; for control cheese 83.12 ± 0.06, 27.06 ± 0.04, 1.43 ± 0.4 respectively with significant difference (*p* < 0.05) were observed. As expected, L* (lightness) value was higher in cheese sample which is more whitish in appearance CC. The higher b* and a* values were observed in CP; implying it has more yellowish and reddish appearance compared to CC. Carotenoid pigments and minor phenolic compounds in pineapple extract may introduce a yellowish tint, potentially increasing the b* value.

**TABLE 10:** Color properties of chickpea cheeses.

Samples	Color properties		
	L*	b*	a*
CP	81.08 ± 0.02^a^	30.02 ± 0.01^a^	1.89 ± 0.01^a^
CC	83.12 ± 0.06^b^	27.06 ± 0.04^b^	1.43 ± 0.4^b^

*Note:* All the values are means ± standard deviations. Means within a column with different letters are significantly different at *p* < 0.05.

Abbreviations: CC, control cheese sample without pineapple extract; CP, optimized cheese sample with pine apple extract.

##### 3.5.2.3 pH Analysis

The introduction of pineapple extract to chickpea cheese significantly (*p* < 0.05) lowered the pH of cheese compared to the control cheese (cheese without extract). This is probably due to the presence of organic acids such as citric acid found in pineapple. The pH of optimized cheese sample CP was ranged 5.73–5.74; which is 5.73 ± 0.01; In contrast; the control cheese sample tends to maintain a higher pH, generally ranging between 6.2–6.5 which is 6.39 ± 0.12; due to the absence of added acids. Reduced pH in pineapple is advantageous offering improved microbial quality and extended shelf life. Pineapple liquid extract fortified curd reduces the growth of microorganisms than control sample as bacteria are intolerant at low pH (Dutta et al. 2016).

#### 3.5.3 Morphological and Molecular Analysis of Chickpea Cheese

##### 3.5.3.1 Scanning Electron Microscopy (SEM) Analysis

**FIGURE 17:**
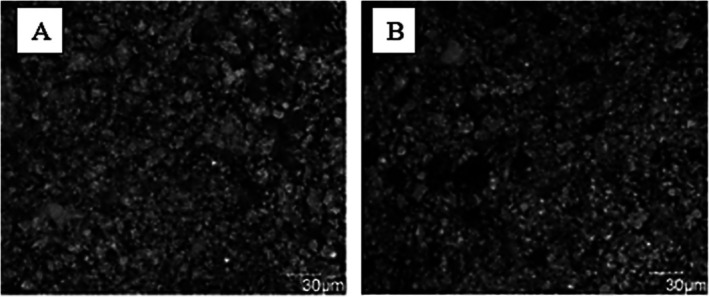
Scanning electron microscopy image of cheese samples at 30 μm magnification level: (A) optimized cheese CP and (B) control chickpea cheese CC.

Cheese microstructure is the spatial arrangement of particles that combine in to a bond and chain to form an overall matrix of proteins dispersed by water, fat, globules, and minerals (Raikos et al. 2018). According to Lamichhane et al. (2020), microscopic visualization of the microstructure enables us to better comprehend fat‐protein interactions within the cheese matrix. Chickpeas primarily contain globular proteins that may form a gel‐like network during coagulation by trapping fat and water. Bromelain from pineapple can modify this protein structure, affecting cheese texture. SEM analysis (Figure 17) showed an increased protein matrix and larger pores in the optimized cheese (CP) compared to the control (CC), likely due to moisture differences, as larger pores facilitate whey drainage and reduce moisture. Large pore size in combination with a denser protein networkallows the matrix to retain water effectively while maintaining structure and enhancing the textural properties. The CC sample exhibited larger black regions, suggesting carbohydrate structuring, while fat globules appeared as small spherical and non‐spherical shapes in both samples. However, the overall microstructural differences between CP and CC were minimal.

The pore size for both cheese samples is small, which is probably due to the preparation process; it has been known for a long time that the preparation process can have a great impact on the structure of the cheese (Fox et al. 2017).

##### 3.5.3.2 Fourier‐Transform Infrared (FTIR) Analysis

**FIGURE 18:**
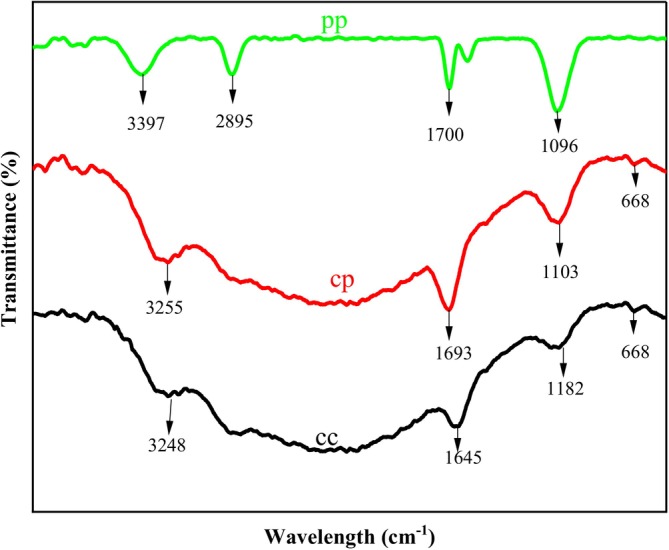
FTIR Spectrum of pineapple extract (PP), optimized cheese (CP), and control cheese (CC).

The FTIR spectra of the cheese samples and pineapple extract are presented and compared in (Figure 18). The spectrum of pineapple extract confirmed that it is rich in organic acids and sugars. The presence of hydroxyl groups from water, sugar, and organic acids is indicated at a broad and strong absorption around 3300 cm^−1^ corresponds to O‐H stretching vibrations; whereas the cheese with pineapple extract retains this band but broadens, indicating hydrogen bonding between pineapple compound and chickpea proteins. On the other hand, the cheese sample without pineapple extract showed N‐H/O‐H from native proteins with no added interaction.

Peaks around 2900 cm^−1^ and 1700 cm^−1^ indicate C‐H stretching and presence of carboxyl groups respectively which is probably from carboxylic acids such as citric acid in pineapple. Cheese with pineapple extract showed reduced intensity in carboxylic group and absence of this group is observed in pure chickpea cheese.

The incorporation of pineapple significantly altered the protein structure of chickpea cheese, as evidenced by changes in the amide bands. This change indicates that the interaction between protein functional groups and polyphenols from pineapple extracthelps preserve protein integrity and bioactive compounds, enhancing nutrient retention.

#### 3.5.4 Microbiological and Nutritional Composition Analysis of Cheeses

##### 3.5.4.1 Microbiological Analysis

The control cheese had the highest total microbial count compared with the optimized cheese sample CP (Table 11). The lowest count observed in the optimized cheese sample was probably the effect of reduced pH, which creates an unfavorable environment for microbial growth, and to the antibacterial activity of bromelain in the pineapple extract. According to (Yoghurt 2016) who used pineapple in the preparation of flavored yogurt, the control yogurt significantly presented the high value of total plate counts, while the treated sample with pineapple had the lowest plate counts. They explained the reduction of bacterial growth to the antibacterial effect of bromelain in pineapple. Similarly, (Hamad et al. 2019) reported that as the pineapple level increased, the counts of St. Thermophiles were decreased in probiotic dairy beverage samples fortified with different ratios (w/w) of pineapple pulp.

**TABLE 11:** Microbial load of chickpea cheese samples.

Samples	Total plate count (CFU/g)
CP	8.4 × 10^4a^
CC	1.6 × 10^5b^

*Note:* All the values are means ± standard deviations. Means within a column with different letters are significantly different at *p* < 0.05.

Abbreviations: CC, control cheese sample without pineapple extract; CP, optimized cheese sample with pineapple extract.

##### 3.5.4.2 Mineral Composition Analysis

Table 12 shows that adding pineapple ( *Ananas comosus* ) extract improved the mineral content of chickpea cheese (CP), notably increasing potassium by 42.4% compared to control (CC), reflecting pineapple's natural potassium content (~180–325 mg/100 g). Slight increases in calcium, magnesium, and iron in CP may result from pineapple's mineral content and bromelain‐enhanced bioavailability. The extract's acidity and ascorbic acid likely improved mineral solubility and iron absorption, making CP a nutritionally superior product with enhanced mineral content and bioavailability making the cheese a functional food. Regular consumption could offer added health benefits; support bone health, electrolyte balance, and overall nutrient intake, aligning with consumer interest in foods that promote well‐being and provide natural, bioactive compounds.

**TABLE 12:** Mineral contents of chickpea cheeses.

Parameters (mg/100 g)	CP	CC
Calcium	57.48 ± 0.8^a^	56.03 ± 0.03^b^
Magnesium	45.18 ± 0.03^a^	44.02 ± 0.06^b^
Iron	3.28 ± 0.01^a^	3.25 ± 0.01^a^
Potassium	12.95 ± 0.06^a^	9.03 ± 0.02^b^

*Note:* Means within a row with different letters are significantly different at *p* < 0.05.

Abbreviations: CC, control cheese sample without pine apple extract; CP, optimized cheese sample with pine apple extract.

#### 3.5.5 Sensory Property Analysis of Cheeses

**FIGURE 19:**
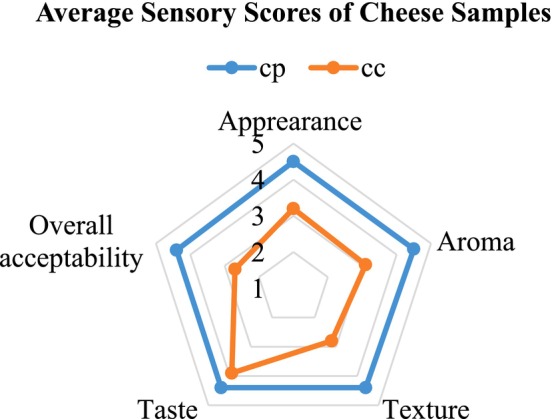
Visual representation of the sensory evaluation of optimized chickpea cheese CP and control cheese CC (cheese coagulated with and without pineapple extract, respectively) across five attributes (appearance, flavor, color, texture, and overall acceptability).

A 5‐point hedonic scale was employed to evaluate chickpea cheese with and without pineapple extract (control) based on appearance, aroma, texture, taste, and overall acceptability. As shown in the radar graph (Figure 19), the optimized cheese scored higher in appearance, likely due to its uniform texture (Figure 15). Taste scores slightly differed, with the optimized cheese having a more pronounced fruity flavor (*p* > 0.05), compared to the beany taste of the control. Similarly, (Bakar et al. 2024) reported that among all the samples yogurt fortified with 4% of pineapple extract received the highest score in all attributes. Finally, approval of chickpea cheese made with addition of pineapple extract has been revealed with a good acceptability score indicating that consumers would recently buy this product. The optimized cheese was rated higher: 4.5 for Appearance and Aroma; 4.4 for Texture, Taste, and overall acceptability, while the control cheese obtained 3.2 for appearance, 3.1 for aroma, 2.8 for texture, 3.9 for taste, and 2.7 for overall acceptability. The increased textural property observed in CP can be directly linked to the denser protein network, while the yellowish color can be attributed to the carotenoid compounds in pineapple extract.

## 4 Conclusion

This research provides valuable insights into plant‐based cheese development, highlighting its potential for sustainable food production using minimal ingredients. The study demonstrated that chickpea‐based cheese coagulated with pineapple ( *Ananas comosus* ) extract under optimal conditions MER (0.24), CT (40°C), and Ct (58.72 min) achieved superior quality compared to the control. Pineapple ( *Ananas comosus* ) extract significantly improved cheese yield (27.54% ± 0.1% vs. 18.89% ± 0.13%), and enhanced textural properties such as hardness (24.22 to 35.04 N), cohesiveness (0.29 to 0.57 N), and adhesiveness (1.68 to 2.14 N). Nutritional quality was also improved, notably in potassium (12.95 vs. 9.03 mg/100 g), magnesium, and calcium content. Sensory evaluation confirmed better scores for appearance, aroma, texture, taste, and overall acceptability in the optimized cheese (rated 4.4–4.5) compared to the control (rated 2.7–3.9). Overall, incorporating pineapple extract into chickpea milk presents a promising approach to producing acceptable and nutritious plant‐based cheese. Further research is recommended on by‐products pineapple (slush and leaves) and utilization of chickpea cake residue and whey.

The developed cheese showed promising nutritional, textural, and microbial properties. However, the study was limited by single‐point microbial assessment, lack of shelf‐life and stability data, and incomplete nutritional profiling such as vitamins, amino acids, anti‐nutrients, and bioactive peptides; industrial scalability was also not assessed. These aspects should be addressed in future studies to enhance product optimization and commercial applicability.

## Author Contributions

**Lamrot Damene Alemayehu:** conceptualization, methodology, software, validation, formal analysis, investigation, resources, data curation, writing – original draft, visualization. **Eskindir Getachew Fentie:** conceptualization, methodology, validation, investigation, resources, writing – review and editing, data curation, visualization, supervision, project administration. **Henock Woldemichael Woldemariam:** conceptualization, methodology, validation, resources, writing – review and editing, data curation, visualization, supervision, project administration.

## Conflicts of Interest

The authors declare no conflicts of interest.

## Data Availability

Data are available from the corresponding author upon request.
